# Supplementary material for: Single-shot electron radiography using a laser–plasma accelerator
Source: Sci Rep. 2023 Feb 8;13:2227. doi: 10.1038/s41598-023-29217-4 (PMC9908895; doi:10.1038/s41598-023-29217-4)
Supplement: Supplementary file 1 — Supplementary Information. [file 41598_2023_29217_MOESM1_ESM.docx]

**Appendix A**

The electron energy spectrum has been extensively measured in previous experiments with this platform and several representative spectra are shown in Fig 1A. This sample has two “typical” results (samples 1 and 2) and one low energy outlier (sample 3) to show the potential variation.

**Figure 1A.** Several sample electron energy spectra from the OMEGA EP generated electron beam

The weighted average energy is 20 MeV with an average energy variation of $\pm5$ MeV, but there are outliers that exceed this range on some shots (such as sample 3). The energy spread and variation in weighted average electron energy makes the use of this beam for field measurements challenging and motivates further upgrades to the system to generate a more monochromatic beam with greater reliability in generating the same peak energy.

**Appendix B**

To derive Eq. 7, one first starts with Eq. 2 for electric rigidity.

$E\rho= \frac{pc\beta}{q}$ (1A)

Using the mean-particle momentum of the beam, a radius of curvature from a given electric-field relationship is established. We can then solve for electric field and achieve the following:

$E= \frac{pc\beta}{q\rho}$ (2A)

We then treat the change in momentum and curvature as small changes by assuming the radiography object is acting as a very thin lens compared to the distances involved. The term $\delta\rho$ will also be changed to $\delta x$ to eliminate confusion with the term $\delta p$.

$E\approx\frac{\delta p}{\delta x}\frac{c\beta}{q}$ (3A)

Fig. 2A shows the basic geometry of the derivation, where *y* is the distance from source to radiography object, *x* is the distance from the radiography object to the imaging plane, *r* is the radius of the radiography object, *R* is the expected radius of the image, and $R'$ is the actual radius of the image seen.

**Figure 2A.** Outline of radiography geometry for electric-field alterations of the image

Using Fig 2A the radius of curvature of the beam can be derived by first determining the angle between the unaffected trajectory (R) and the new trajectory (R’) utilizing the trapezoid created by *r*, *x* and *R’*.

Utilizing this geometry and the thin lens approximation and the geometry in Fig 2A we can then determine the change in *p* as seen in Eq 4A

$\delta p= \left( p^{'}-p \right)=p_{x}\left( \frac{R^{'}-R}{x} \right)$ (4A)

Further using the geometry of the system, we can then turn the x component of the momentum into Eq 5A

$p_{x}\left( \frac{R^{'}-R}{x} \right)=p\left( \frac{y}{\sqrt{y^{2}+r^{2}}} \right)\left( \frac{R^{'}-R}{x} \right)$ (5A)

Inserting this back into Eq 3A then generates Eq. 7 from the main manuscript.

$E=\left( \frac{y}{\sqrt{y^{2}+r^{2}}} \right)\left( \frac{R^{'}-R}{x} \right)\frac{pc\beta}{\delta xq}$ (6A)

**Appendix C**

Basic simulations of the electron beam radiography experiments were generated using the software G4 Beamline [37]. The simulations are limited to <10^7^ electrons due to computational limits, which causes the resulting images to be less clear than the actual radiographs due to aliasing. These simulations are also limited by their simple beam geometry and lack of complex electric field gradients. They were only used to guide investigation of field induced magnification changes, rather than confirm particular electric field values for a given experiment. To test the effect of a simple radial electric field, simulations were generated with a W target in the 3.58 cm configuration and radial electric fields ranging from 0 GV/m to 2 GV/m. The change in the image was then measured (Fig 3A) and the effect on the image determined.

**Figure 3A.** The simulated radiograph of a W test object at 3.58 cm from the source (a) without an electric field and (b) with a 1.5 GV/m radial electric field. The yellow line is representative of the measurement of the target radius in the simulated images and are ~3 mm difference in length.

Magnetic fields, electric fields of opposite sign, electric fields along the thickness of the target and combinations of the options were all tested. It was found that only radial electric fields on the order of 1-3 GV/m matched what was seen in the experiments best (Fig 4A). Magnetic fields both rotated and distorted the image, while lengthwise electric fields provided effectively no change in the image. Combinations of electric and magnetic fields could not prevent visual obvious image distortion and thus, are not considered likely.

**Figure 4A.** Plot of measured radius of target in G4 Beamline simulation vs radial electric field strength
